# Enhancing & Mobilizing the POtential for Wellness & Emotional Resilience (EMPOWER) among Surrogate Decision-Makers of ICU Patients: study protocol for a randomized controlled trial

**DOI:** 10.1186/s13063-019-3515-0

**Published:** 2019-07-09

**Authors:** Holly G. Prigerson, Martin Viola, Chris R. Brewin, Christopher Cox, Daniel Ouyang, Madeline Rogers, Cynthia X. Pan, Shayna Rabin, Jiehui Xu, Susan Vaughan, Janna S. Gordon-Elliot, David Berlin, Lindsay Lief, Wendy G. Lichtenthal

**Affiliations:** 1 Center for Research on End-of-Life Care, Weill Cornell Medicine, New York, NY USA; 2 Department of Medicine, Weill Cornell Medicine, New York, NY USA; 3 Department of Clinical, Educational and Health Psychology, University College London, London, UK; 4 Department of Medicine, Division of Pulmonary & Critical Care Medicine, Duke University, Durham, NC USA; 5 NewYork-Presbyterian Queens, New York, NY USA; 6 Department of Psychiatry, Columbia College of Physicians and Surgeons, New York, NY USA; 7 Department of Psychiatry, Weill Cornell Medicine, New York, NY USA; 8 Department of Psychiatry and Behavioral Sciences, Memorial Sloan Kettering Cancer Center, New York, NY USA

**Keywords:** Critical illness, Psychological distress, Peritraumatic distress, Medical decision-making, Communication, Surrogate decision-makers, Caregivers

## Abstract

**Background:**

Critical illness increases the risk for poor mental health outcomes among both patients and their informal caregivers, especially their surrogate decision-makers. Surrogates who must make life-and-death medical decisions on behalf of incapacitated patients may experience additional distress. EMPOWER (Enhancing & Mobilizing the POtential for Wellness & Emotional Resilience) is a novel cognitive-behavioral, acceptance-based intervention delivered in the intensive care unit (ICU) setting to surrogate decision-makers designed to improve both patients’ quality of life and death and dying as well as surrogates’ mental health.

**Methods:**

Clinician stakeholder and surrogate participant feedback (*n* = 15), as well as results from an open trial (*n* = 10), will be used to refine the intervention, which will then be evaluated through a multisite randomized controlled trial (RCT) (*n* = 60) to examine clinical superiority to usual care. Feasibility, tolerability, and acceptability of the intervention will be evaluated through self-report assessments. Hierarchical linear modeling will be used to adjust for clustering within interventionists to determine the effect of EMPOWER on surrogate differences in the primary outcome, peritraumatic stress. Secondary outcomes will include symptoms of post-traumatic stress disorder, prolonged grief disorder, and experiential avoidance. Exploratory outcomes will include symptoms of anxiety, depression, and decision regret, all measured at 1 and 3 months from post-intervention assessment. Linear regression models will examine the effects of assignment to EMPOWER versus the enhanced usual care group on patient quality of life or quality of death and intensity of care the patient received during the indexed ICU stay assessed at the time of the post-intervention assessment. Participant exit interviews will be conducted at the 3-month assessment time point and will be analyzed using qualitative thematic data analysis methods.

**Discussion:**

The EMPOWER study is unique in its application of evidence-based psychotherapy targeting peritraumatic stress to improve patient and caregiver outcomes in the setting of critical illness. The experimental intervention will be strengthened through the input of a variety of ICU stakeholders, including behavioral health clinicians, physicians, bereaved informal caregivers, and open trial participants. Results of the RCT will be submitted for publication in a peer-reviewed journal and serve as preliminary data for a larger, multisite RCT grant application.

**Trial registration:**

ClinicalTrials.gov, NCT03276559. Retrospectively registered on 8 September 2017.

**Electronic supplementary material:**

The online version of this article (10.1186/s13063-019-3515-0) contains supplementary material, which is available to authorized users.

## Background

Critical illness increases the risk for poor mental health outcomes among both patients [1] and families [2]. The burden of psychological distress may be especially great for the surrogate decision-makers of intensive care unit (ICU) patients who are unable to adequately communicate their treatment decisions due to factors including altered consciousness, requirement for invasive life support, or the severity of their underlying illness. This leaves surrogate decision-makers in the challenging situation of potentially needing to make life-and-death decisions without the patient’s input about treatment preferences at a time while they themselves are significantly distressed. Given that ICU surrogates are at heightened risk for poor psychological outcomes [3, 4], there have been calls for interventions that can help ICU surrogates cope throughout the illness course, from ICU admission through discharge or bereavement, in order to improve surrogate mental health [5].

Past efforts to address these challenges have so far produced disappointing results for improving end-of-life (EoL) care and surrogate mental health; moreover, some psychosocial interventions may carry risk. In a recent randomized controlled trial (RCT), post-traumatic stress disorder (PTSD) symptoms increased for surrogate decision-makers of ICU patients who received a family meeting intervention led by palliative care medical specialists that was designed to reduce surrogate anxiety and depression [6]. Another intervention designed to improve mental health outcomes through sending handwritten condolence cards to relatives of patients who died in the ICU was shown to worsen depression and PTSD symptoms [7]. A web-based, personalized decision aid for surrogates of ICU patients did not reduce surrogates’ symptoms of depression, anxiety, or PTSD or change clinical outcomes compared to usual care [8]. Finally, a multicomponent, nurse-led intervention designed to reduce depression, anxiety, and PTSD focused on the provision of emotional, communication, decisional, and anticipatory grief support for ICU family caregivers, but did not utilize targeted strategies to reduce clinical symptoms [9, 10]. In fact, the investigative team specifically noted that the interventionists did not have advanced training in patient counseling [9], and, similar to other trials, the results showed no significant effects on the primary mental health outcomes of anxiety or depression, or on secondary outcomes of PTSD [11].

The primary limitation of these interventions is that although they targeted longer-term (e.g., 3 and 6 months post intervention) mental health outcomes, they were not explicitly designed using empirically supported psychological treatments to address clinically significant mental health symptoms or to provide coping skills that could be applied beyond the ICU stay. Furthermore, they were not delivered by trained mental health clinicians. In fact, White et al. [5] concluded in response to the study findings that a brief, “psychologically focused intervention” should be developed and tested.

To address these limitations, we propose to target surrogate decision-maker mental health as a way to both improve surrogates’ capacity to cope with the stress of the patient’s ICU stay and also improve decision-making on the patient’s behalf. We propose to develop, refine, and evaluate EMPOWER (Enhancing & Mobilizing the POtential for Wellness & Emotional Resilience), a very brief mental health intervention for surrogate decision-makers of ICU patients who are unable to communicate their EoL care preferences. Delivered by a trained mental health professional in the ICU setting, EMPOWER is theoretically grounded in cognitive-behavioral and acceptance-based therapies. EMPOWER aims to improve surrogate mental health outcomes, increase rates of advance care planning (e.g., rates of Do Not Resuscitate (DNR) orders or advanced directive completion), promote value-concordant care through clarifying surrogate perceptions of incapacitated patients’ treatment preferences, improve patient quality of life/death as perceived by the surrogate, and reduce surrogate decisional regret about the patient’s ICU care.

## Methods

### Overview

The methods for the EMPOWER study were developed in accordance with the SPIRIT guidelines [12] (Additional file 1). Any prospective amendments to the protocol, eligibility, or outcomes will first be approved by the institutional review boards of the study sites.

#### Key study objectives

1. Develop EMPOWER for surrogate decision-makers of critically ill ICU patients who are unable to make medical decisions. Key informants, including bereaved informal caregivers of ICU patients and clinicians, will be asked to evaluate the EMPOWER intervention manual to increase its potential tolerability, acceptability, and efficacy.
2. Determine the feasibility, tolerability, acceptability, and preliminary effects of EMPOWER on surrogate mental health. We hypothesize that the revised EMPOWER intervention will be feasible, tolerable, and acceptable. The primary outcome will be symptoms of peritraumatic distress measured following the intervention compared to enhanced usual care. Additional outcomes at 1-month and 3-month follow-up from post-intervention assessment will be compared to enhanced usual care as well.
3. Estimate the effects of EMPOWER on patient outcomes in the months following the post-intervention assessment. Patients who receive EMPOWER are hypothesized to have higher rates of engagement in advance care planning (e.g., a DNR order completed), better surrogate-reported quality of life/quality of death, and more value-concordant care (measured by comparing intensity of care at EoL to surrogate perception of patient treatment preferences) compared to patients whose surrogates receive enhanced usual care.

### Trial design

**Fig. 1:**
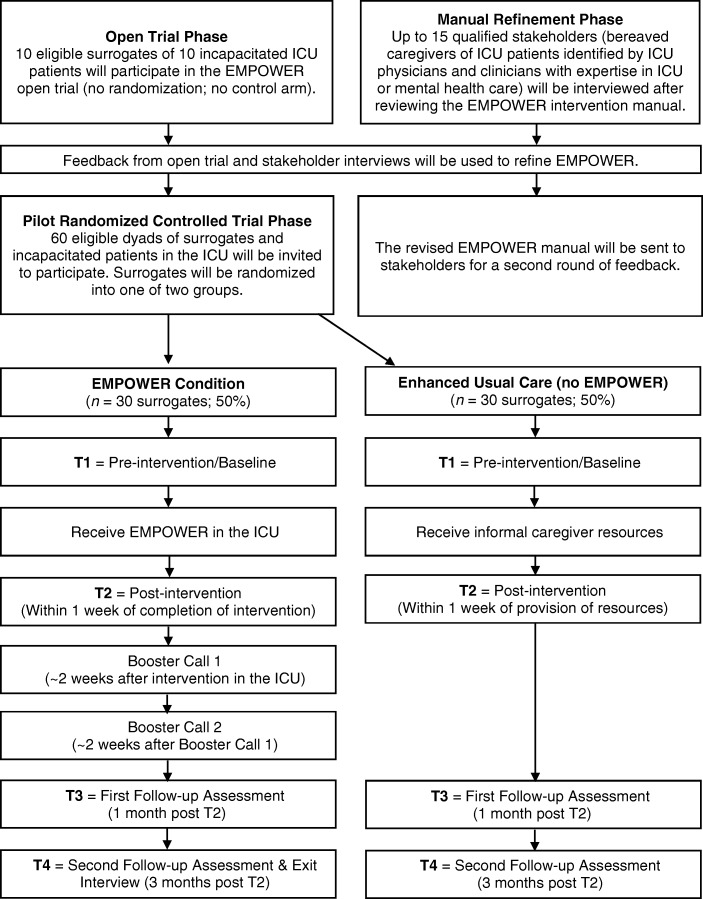
EMPOWER timeline. EMPOWER Enhancing & Mobilizing the POTential for Wellness & Emotional Resilience among Surrogate Decision-Makers of ICU Patients, ICU intensive care unit

The EMPOWER study is comprised of two phases conducted simultaneously in preparation for the subsequent RCT. A timeline of the EMPOWER project is presented in Fig. 1. Phase 1 will first involve both an open trial, enrolling 10 surrogate decision-makers who will receive the EMPOWER intervention, and provide feedback about administration of EMPOWER. The concurrent manual refinement activities will involve obtaining feedback on the EMPOWER intervention manual itself from 15 stakeholders (bereaved informal caregivers and ICU clinicians or mental health clinicians). Feedback from both the open trial and stakeholder interviews will then be used to refine the EMPOWER intervention.

Phase 2 will involve a multicenter, open-label, parallel-group, exploratory RCT, which aims to enroll up to 60 eligible surrogates of 60 incapacitated patients in the ICU. This sample size ensures stable estimates of treatment effects and confidence intervals, and, in case the effects of EMPOWER happen to be large, adequate (~ 80%) statistical power to detect a minimum treatment effect size (Cohen’s *d*) of 0.75 (at α = 0.05). Surrogates will be block-randomized to either the EMPOWER intervention or enhanced usual care and will complete self-report measures (*n* = 30 in each group). A usual care comparator will be enhanced with a packet providing general information and recommendations on serving as an informal caregiver from the National Alliance for Caregiving (http://www.caregiving.org/pdf/resources/CFC.pdf) as well a handout documenting site-specific resources for caregivers at each hospital. We will document the availability and use of social support services provided within each of the three participating ICU sites to control for inter-institutional variability on the provision of supportive services as usual care.

### Location and participants

The study will take place at NewYork-Presbyterian Hospital/Weill Cornell Medical Center, NewYork-Presbyterian Queens, and Memorial Sloan Kettering Cancer Center in New York City. Research assistants will screen potential patient and informal caregiver dyads, and consent them as research participants following approval from ICU physicians and guidance from allied health staff. This study will involve ICU clinicians, patients, and patients’ surrogates as participants as well as stakeholders. Feedback from patients and informal caregivers is integrated into several stages of the EMPOWER trial. Bereaved informal caregivers of ICU patients and ICU clinicians will be consulted to improve the EMPOWER intervention. Additionally, participants in the open trial will be consulted through exit interviews to share their suggestions in improving the intervention, assessments, and recruitment procedures of the trial. All research participants will be compensated to promote retention and complete follow-up. Participating stakeholders will receive compensation of a $50 gift card after reviewing the manual and providing feedback. Participating surrogates in the open trial and RCT will receive $25 following completion of each assessment and exit interview.

### Eligibility criteria

Inclusion criteria for open trial and RCT participants:

1. Patients (age > 21 years) in the ICU/step-down units who cannot communicate treatment preferences, as determined by ICU physicians or fellows, and whose ICU physicians or fellows would not be surprised if the patient did not survive more than 3 months.
2. Informal caregivers of ICU patients whom ICU physicians or fellows indicate as the decision-making surrogate for the patient, or who is listed as such in the patient’s medical record.
3. Surrogates must speak English.
4. Surrogates must either meet the threshold for a high degree of dependence on the patient (determined by the summed score of the overall dependence and emotional dependence on the patient items of the Partner Dependency Scale [13] as greater than 8) or a high degree of anxiety (determined by scoring greater than 5 on either anxiety item from the McGill Quality of Life Questionnaire [14]).

Exclusion criteria for open trial and RCT participants:

1. Patients and surrogates who do not meet the eligibility criteria or surrogates who endorse suicidal ideation in the past month based on responses to the Columbia Suicide Severity Rating Scale [15].

Inclusion criteria for stakeholders:

1. Bereaved family caregivers of patients treated in the ICU identified by referring clinicians and through support groups, clinics, and word of mouth.
2. Clinicians with expertise in mental health care and/or critical care including, but not limited to, nurses, nurse practitioners, social workers, psychologists, hospital chaplains, psychiatrists, and other physicians.

Exclusion criteria for stakeholders:

1. Bereaved informal caregivers or clinicians who do not meet the eligibility criteria.

### Interventions

The EMPOWER intervention will be administered by trained mental health professionals such as psychologists or social workers. The EMPOWER intervention targets symptoms of peritraumatic stress and anticipatory grief that may interfere with optimal decision-making on the patient’s behalf or lead to adverse health outcomes such as prolonged grief disorder or PTSD following the patient’s death or discharge from the ICU. The EMPOWER intervention seeks to act on these symptoms through the reduction of “experiential avoidance” [16, 17] and teaching of coping skills, empirically supported techniques from cognitive-behavioral therapy [18–20], and acceptance and commitment therapy [21–23] that can be applied during the ICU stay and in the immediate aftermath of the ICU stay. It consists of six discrete modules that take approximately 15–20 min each to complete (for an approximate total of 1.5–2 h) and can be delivered flexibly to accommodate the numerous interruptions and unexpected crises typical in an ICU setting. The EMPOWER modules include empathetic listening and alliance building, breathing retraining, grounding exercises, guided mindfulness meditation, psychoeducation about cognitive-behavioral and acceptance-based coping strategies, invoking of the patient’s voice through an imaginal dialogue, and coping rehearsal to prepare for potentially distressing scenarios. A brief summary of the structure of the intervention is presented in Table 1. The six modules can be delivered in the ICU in a single session or in multiple brief sessions based on the surrogate’s preference. Following the initial EMPOWER session conducted in the ICU, two booster sessions will be delivered by phone 2 and 4 weeks after the end of the intervention. Booster sessions will focus on issues relevant to the surrogate, such as bereavement, and reviewing the skills taught in the original session to coping with new challenges. Each booster session will last approximately 45–60 min. Of note, the content and format of EMPOWER will be further developed through the input of surrogates in the open trial, bereaved informal caregivers who have had relevant experiences in the ICU, and clinicians with expertise in mental health and/or critical care.

**Table 1:**
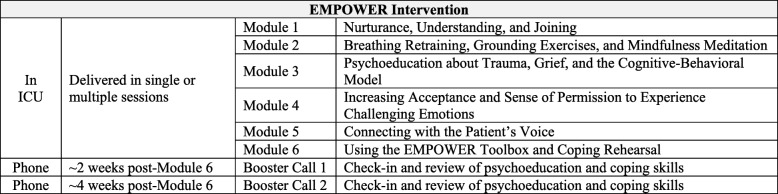
The EMPOWER intervention

*EMPOWER* Enhancing & Mobilizing the POtential for Wellness & Emotional Resilience, *ICU* intensive care unit

EMPOWER will be delivered by at least a master’s-level mental health clinician interventionist who will receive intensive training prior to delivering the intervention and regular supervision after each session. The interventionists will communicate with the medical team as needed, but will provide a safe space separate from the ICU clinicians without any agenda about the patient’s care. While a multidisciplinary approach in the ICU is invaluable, anecdotal evidence suggests that interpersonal dynamics between the surrogates and the medical team sometimes complicate surrogates’ ability to independently consider their and the patient’s wishes (e.g., surrogates have reported feeling pressured by hospital staff to sign a DNR order while the patient is in the ICU, and they feel conflicted and/or defensive about this request). Sessions will be audio recorded (or video recorded if the surrogate provides permission) so that treatment fidelity can be regularly monitored and independently rated by trained research assistants. We will monitor the progress of all participants and request their continued participation in the EMPOWER intervention. If a participant has not completed part of the intervention, we will contact that participant to remind and encourage them to continue up to three times, with an upper limit of contact in place to prevent bothering participants.

Enhanced usual care will consist of the various interactions a surrogate may have with clinicians in the ICU, which may include social work and chaplaincy staff who serve as providers of psychosocial support. Additionally, a packet providing information about informal caregiving and resources will be provided to surrogates in the control group by research staff. Lastly, a referral list of site-specific resources such as caregiver support groups and hotlines will be provided. Use of the various components of enhanced usual care will be monitored and extensively tracked through review of notes in the patient’s medical record and surrogate self-report. Enhanced usual care was chosen as the comparator in this study in order to determine whether the EMPOWER intervention serves as an effective support for surrogates above and beyond standard practice. Having three sites, each with unique practices for supporting informal caregivers and surrogates, will allow the intervention to be compared to multiple smaller subsets of standard treatment, and at the same time also reflect general psychosocial informal caregiver support.

Surrogate decision-makers will be permitted to continue to see any outside mental health professionals during the trial. Mental health treatment they receive from outside professionals, as reported to study clinicians by the subjects, will be documented and controlled for during data analysis.

### Assignment of interventions

Participants in the open trial will all be assigned to the EMPOWER intervention. Participants in the RCT will be randomized to either EMPOWER or the control group with a block randomization procedure in REDCap [24] using computer-generated random numbers generated in R Studio [25]. Research assistants will randomize a participant using REDCap following the participant’s completion of the consent, eligibility screener, and baseline assessment. Because a co-principal investigator will be conducting supervision for the interventionists, and because different assessments will be administered depending on the intervention assignment, the only person completely blinded to group assignment will be the data analyst/statistician.

### Outcomes

1. The first goal of this study is to determine the feasibility and acceptability of the EMPOWER intervention. These outcomes will be measured quantitatively within the week following the intervention (T2) through a post-intervention questionnaire and at 1-month (T3) and 3-month (T4) follow-up from post-intervention assessment (including a qualitative exit interview at T4 of study participants who were assigned to the EMPOWER intervention arm). More specifically, these assessments will measure participant-perceived helpfulness/satisfaction to determine acceptability. Tolerability will be measured in these assessments through participant reports of negative experiences, emotional difficulties, and perceived costs and benefits of participating in the intervention.
  Targets will include completion of 4/6 modules for feasibility, and for acceptability an average response score of at least 4 to items 1, 3, and 7 of the post-intervention satisfaction questionnaire among at least 60% of intervention recipients. Rates of recruitment, reasons for refusal, number of modules/booster calls completed, and study attrition will also be examined. Drop-out post intervention will not be considered a metric of tolerability due to the highly stressful and variable circumstances (e.g., bereavement) of ICU caregiving, unless participants drop out of the study and specifically express that they consider it to be too distressing.
2. The EMPOWER study also aims to improve surrogates’ symptoms of psychological distress. This will be measured by comparing the EMPOWER group to the enhanced usual care group at multiple time points. The primary outcome will be in peritraumatic distress at post-intervention assessment (T2), administered within a week of the intervention. Secondary outcomes will be differences in symptoms of PTSD, prolonged grief disorder, and experiential avoidance, and exploratory outcomes will be anxiety, depression, and decisional regret at 1-month and 3-month follow-up from post-intervention assessment (T3 and T4).
3. Additionally, the EMPOWER study aims to improve patient outcomes through promoting value-concordant care, quality of life, and quality of death. Rates of value-concordant care will be measured through comparing surrogate perceptions of patient treatment preferences assessed at baseline (e.g., a preference to prioritize care focused on quality of life over quantity of life) with the intensity of care provided in the indexed ICU stay (e.g., indication of cardiopulmonary resuscitation, dialysis, mechanical ventilation, chemotherapy, or parenteral nutrition, and palliative care in the medical record). We will compare surrogate-assessed patient quality of death (for patients who died) using the CEQUEL [26] between groups, measured at either T3 or T4, depending on which time point first follows the patient’s death. Surrogate-assessed patient quality of life will be assessed as relevant to the most recent week (or week alive) through Likert-type items previously published [27], as well as through a revised version of the CEQUEL, and will be measured at 1-month follow up (T3), 3-month follow up (T4), both, or neither, depending on patient status.

### Measures

#### Demographics

Surrogate decision-makers will be asked in a baseline assessment, occurring either in the clinic or over the telephone, their own and the patient’s age (years), gender, race, education, mental health history, income, marital status, religious/spiritual beliefs, advance care planning knowledge/understanding, treatment preferences, and prognostic understanding and their relationship with the patient. Stakeholders will report on their own demographics.

#### Medical factors for patients

We will abstract the medical chart to record patients’ primary hospital and ICU admitting diagnoses (e.g., stage IV pancreatic or NSCL cancer), Do Not Resuscitate/Do Not Intubate order status, advance care planning items (e.g., Living Will, Health Care Proxy, Health Care Power of Attorney), palliative care consultations, and care plans obtained from the medical chart or ICU physicians and fellows. This information will be compiled as a medical chart abstraction and matched with surrogate-assessed patient treatment preferences assessed at baseline to create a measure of rates of value-concordant care. These medical factors, in addition to the CEQUEL [26], will serve to measure the outcomes specified in Objective #3 (see Data analysis plan).

#### Psychosocial factors for surrogate decision-makers

**Fig. 2:**
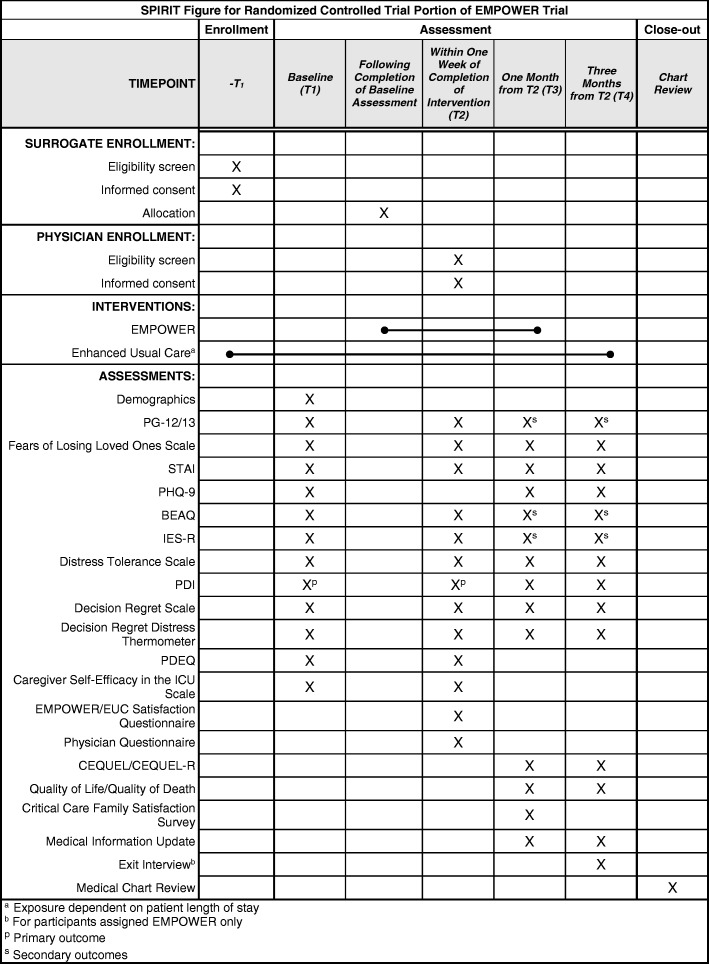
Standard Protocol Items: Recommendations for Interventional Trials (SPIRIT) figure. BEAQ Brief Experiential Avoidance Questionnaire, CEQUEL caregiver evaluation of the quality of end-of-life care, EMPOWER Enhancing & Mobilizing the POtential for Wellness & Emotional Resilience, EUC, ICU intensive care unit, IES-R Impact of Events Scale—Revised, PDEQ Peritraumatic Dissociative Experiences Questionnaire, PDI Peritraumatic Distress Inventory, PG-12/13 Prolonged Grief Disorder-12/13, PHQ-9 Patient Health Questionnaire, STAI State Trait Anxiety Questionnaire

A description of each quantitative measure used at each assessment is provided in Table 2, and a timeline of assessments is provided in Fig. 2.

**Table 2:**
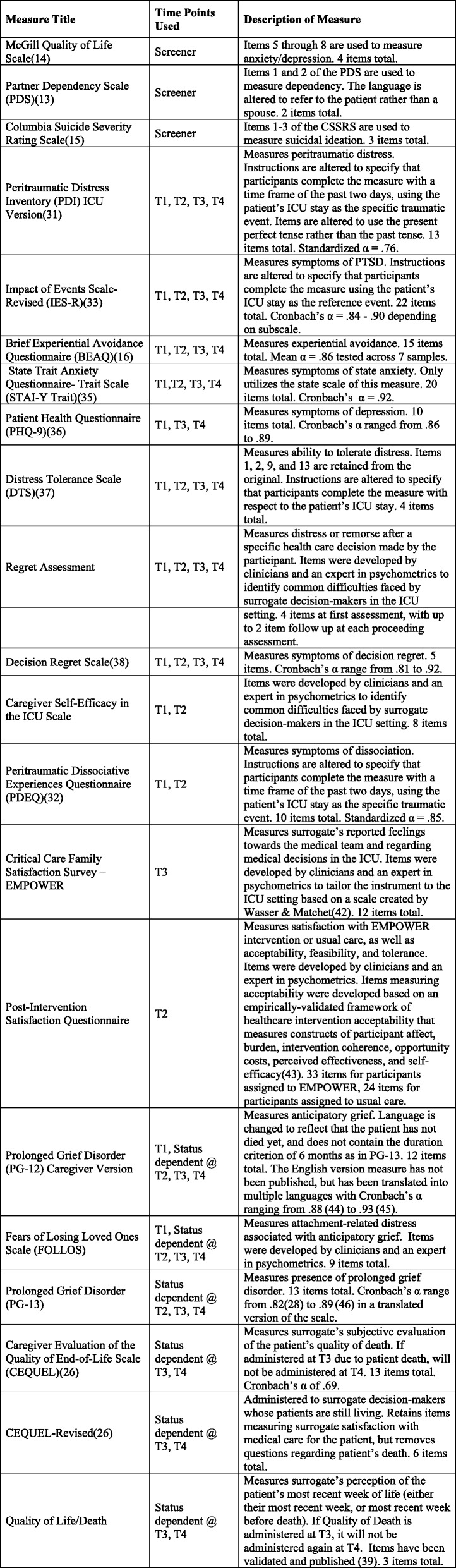
List of assessments

*CSSRS* Columbia Suicide Severity Rating Scale, *EMPOWER* Enhancing & Mobilizing the POtential for Wellness & Emotional Resilience, *ICU* intensive care unit, *PTSD* post-traumatic stress disorder, *T1* pre-intervention/baseline assessment, *T2* post-intervention assessment administered within 1 week of intervention, *T3* 1-month follow-up from post-intervention assessment, *T4* 3-month follow-up from post-intervention assessment

#### Screener

The screener consists of four items from the McGill Quality of Life Questionnaire [14], two items from the Partner Dependency Scale [13], and three items from the Columbia Suicide Severity Rating Scale [15]. We will also obtain the surrogate’s physician/healthcare provider information at baseline should a medical or mental health emergency arise.

#### Pre-intervention/baseline assessment (T1)

Psychiatric history, demographics, and treatment preferences; Prolonged Grief Disorder (PG-12) Caregiver Version [28–30]; Fears of Losing Loved Ones Scale (FOLLOS); Peritraumatic Distress Inventory (PDI) [31]; Peritraumatic Dissociative Experiences Questionnaire (PDEQ) [32]; Impact of Events Scale—Revised (IES-R) [33]; Brief Experiential Avoidance Questionnaire (BEAQ) [34]; State Trait Anxiety Questionnaire—Trait Scale (STAI-Y Trait) [35]; Patient Health Questionnaire (PHQ-9) [36]; Distress Tolerance Scale (DTS) [37] revised version; Caregiver Self-Efficacy in the ICU Scale; and Decision Regret Scale (DRS)—EMPOWER [38].

#### Post-intervention assessment (T2) administered within 1 week of intervention

PG-12 (if patient is alive); FOLLOS (if patient is alive); PG-13 (if patient is deceased) [28–30]; PDI; PDEQ; IES-R; BEAQ; STAI-Y Trait; DTS revised version; Caregiver Self-Efficacy in the ICU Scale; DRS—EMPOWER; and Post-Intervention Satisfaction Questionnaire (PISQ).

#### One-month follow-up from post-intervention assessment (T3)

PG-12 (if patient is alive); FOLLOS (if patient is alive); PG-13 (if patient is deceased); PDI; IES-R; BEAQ; STAI-Y Trait; DTS revised version; CEQUEL-R (if patient is alive) [26]; CEQUEL [26] (if patient is deceased); quality of life (if patient is alive) [39]; quality of death (if patient is deceased) [39]; DRS—EMPOWER; and medical information update.

#### Three-month follow-up from post-intervention assessment (T4)

PG-12 (if patient is alive); FOLLOS (if patient is alive); PG-13 (if patient is deceased); PDI; IES-R; BEAQ; STAI-Y Trait; PHQ-9; DTS revised version; Critical Care Family Satisfaction Survey— EMPOWER; CEQUEL-R (if patient is alive); CEQUEL (if patient is now deceased, but was alive at T3); quality of life (if patient is alive); quality of death (if patient is deceased); DRS—EMPOWER; medical information update; and qualitative exit interview (for patients receiving the EMPOWER intervention only).

#### Qualitative data

Surrogates will provide feedback on the intervention in a post-intervention satisfaction questionnaire at T2 and a one-on-one semi-structured exit interview at T4 conducted over the phone or in person solely for participants assigned to the experimental arm. Stakeholders will provide feedback on the intervention manual in self-report questionnaires, written form, and/or in-person interviews.

If participants drop out of the study, the investigative team will attempt to ask them for their reasons for ceasing to participate, but no further data will be collected.

### Data analysis plan

The following are descriptions of the statistical procedures performed to test each of the hypotheses. Participant data will be stored in a locked file cabinet and using a secured REDCap database. Missing data will be estimated using a multiple imputation procedure described by Schafer and Olsen [40]. There will not be a data monitoring committee due to the trial’s relatively short duration and the minimal risks that the intervention poses. Trial data will not be independently audited. An interim analysis of the pilot data will occur to inform the conduct of the RCT and edits to the EMPOWER manual.

#### Objective #1: refine EMPOWER for surrogate decision-makers of critically ill patients who are unable to communicate in the ICU

We will use thematic content analysis, a well-established, systematic qualitative analysis approach in health research, to identify themes from stakeholder participants’ narratives and exit interviews. We will follow Morse’s guidelines for conducting rigorous qualitative research (e.g., audit trail, saturation) [41–46] using Atlas.ti software. We will independently review each interview transcript as well as qualitative data gathered from manual edits and Delphi survey responses, and will synthesize and interpret participants’ feedback about the content of the EMPOWER manual.

#### Objective #2: determine the feasibility, acceptability, tolerability, and preliminary effects of EMPOWER on surrogate mental health

We will compute descriptive statistics to characterize the feasibility and acceptability of EMPOWER by examining helpfulness/satisfaction ratings, rates of recruitment, reasons for refusal, and number of modules/booster calls completed. These will be used to determine whether the EMPOWER intervention meets the targets detailed earlier in the outcomes. Qualitative data analysis will be used to analyze data from open-ended questions to identify the most helpful components of EMPOWER.

To evaluate the preliminary effects of EMPOWER on peritraumatic stress at post-intervention assessment (T2) in the RCT, we will use a hierarchical linear modeling (HLM) and an intent-to-treat approach. HLM is statistically appropriate because it corrects for clustering within interventionists and within surrogates by modeling them as random effects. This will also provide a treatment assignment model coefficient and effect size estimate for our future, larger study.

HLM modeling will determine differences between surrogates and patients assigned to EMPOWER vs enhanced usual care to examine the primary, secondary, and exploratory outcomes described earlier. HLM models will include covariates, either as fixed effect or time varying (e.g., patient death), if those variables are found to be significantly statistically associated with both the intervention assignment and the outcome examined.

#### Objective #3: examine the effects of EMPOWER on patient outcomes in the month following ICU admission

Logistic regression models will regress patient quality of life or quality of death (depending on whether the patient survives or dies in the observation period) for EMPOWER versus the enhanced usual care condition. Logistic regression analyses will model the effects of EMPOWER on the odds of patients’ receipt of value-concordant care (i.e., surrogate baseline assessment of patient preferences regarding quality of life versus quantity of life matched with receipt of intensive life-prolonging procedures/palliative care). Potential differences in assessment timing between groups will be adjusted for.

### Adverse reactions and events

We anticipate that there may be questions in the interview that some study participants find upsetting. However, since study items and topics were chosen to reflect what are likely to be existing concerns, the present study is not expected to markedly increase participants’ psychological distress above their routine concerns. Topics covered during the intervention sessions may be emotional, but related distress is expected to be transient and will be supported by a mental health clinician. In addition, experienced personnel trained in interviewing medically ill individuals and their families will administer all instruments and will be supervised by the study principal investigators (PIs). If a participant assigned to the EMPOWER intervention wishes to stop participating in the intervention for any reason, we will request that they inform the researchers, and, if willing and able, inform the research team of the reason for ceasing participation. If a participant appears to be at risk for harming himself/herself or others during the course of the trial, the researchers will take immediate action to address this risk and the participant would become ineligible for continuing with the study.

Potential adverse events for this project are expected to be all non-physical in nature. The principal investigators will report unanticipated and serious adverse events to the IRB in a timely manner on an ongoing basis. For the purpose of this study, a serious adverse event is defined as an event that, as a direct result of the study, causes serious harm to the participant (e.g., that involvement in the study caused the death of or serious injury to the participant). Adverse events are also reported as part of the progress reports in the non-competitive and competitive renewals for the National Institutes of Health. If at any point during the study period the study intervention is found to be associated with an undue risk for harm to subjects, then the trial will be stopped—such as if the research team determines, in good faith, that the intervention appears to be causing significant emotional distress or impairment for subjects beyond what would be expectable or leading to increased risk for harm to self or others.

All study staff involved in the research are educated on the protection of human research participants and the proposed research will comply with the regulations set forth in 45 CFR Part 46, Protection of Human Subjects. All personnel involved in the proposed protocol have been educated regarding HIPAA regulations and fully understand their responsibility to safeguard the personal health information of every participant involved in the research. Any participant participating in the study may decline to continue participation and may withdraw from the study at any time. Any participant who expresses a desire for more intensive psychosocial support for issues such as PTSD or bereavement following the intervention will receive a customized set of referrals from the study team.

We will collect participants’ medical and mental health history, details about outside clinicians, and emergency contact information. Participants will be screened for suicidality with the Columbia Suicide Severity Rating Scale [15] and in accordance with our screening and management guidelines. If research staff identify signs indicating a significant and acute risk of harm to self or others, such information will immediately be shared with the PIs of the study, so that a plan can be enacted for timely and appropriate assessment and care, provided by a licensed/ board-certified mental health provider or local clinicians (e.g., emergency rooms near the study participant).

Participant confidentiality will only be broken if information gathered during the course of the study indicates that the participant poses a significant and acute risk of harm to self or others. Prior to inclusion in the study, participants will be informed of this exception. If a participant deemed to be at acute risk of self-harm or harm to others cannot be reached by the study team within 3 h (after at least two telephone call attempts and an email requesting a call back), the participant’s emergency contact(s) will be contacted. If an acutely distressed individual who has denied active suicidality or homicidality, but for whom the study team has significant concern, cannot be reached within 24 h (after at least two phone call attempts and an email requesting a call back), the participant’s emergency contact(s) will be contacted. These details are outlined in the informed consent for study participation.

## Discussion

This trial will evaluate the effects of a mental health intervention conducted in the ICU on surrogate decision-makers of incapacitated patients. Psychiatric symptoms of surrogates, participant quality of life and quality of death, and rates of nonbeneficial, burdensome care will be examined.

Previous trials led by ICU and palliative care clinicians have proven inefficacious in improving mental health outcomes in informal caregivers [5–11]. This trial takes a different approach by examining a mental health intervention for mental health problems. Additionally, the EMPOWER intervention will be created and refined based on the input of a variety of ICU stakeholders, including behavioral health clinicians, physicians, and bereaved informal caregivers.

Due to the clinical and logistical aspects of the protocol, the EMPOWER trial will not be blinded. Also, this pilot RCT has limited statistical power. Study participants, however, will be recruited from ICUs across three different hospitals to accelerate recruitment and maximize sample size and diversity. In addition, these data may be used in support of a large-scale, adequately powered study.

If efficacious, the EMPOWER intervention has the potential to improve both the mental health outcomes of informal caregivers and the quality of life at end of life for incapacitated patients receiving intensive care. Through stakeholder feedback, an initial open trial, and an RCT, this pilot study will extensively examine what may potentially serve as an efficient and flexible intervention for incapacitated patients and their surrogate decision-makers in the ICU.

## Trial status

Enrollment has been completed for both open trial and stakeholder feedback. Enrollment for the RCT portion of the EMPOWER trial began in January 2019.

## Additional file

Additional file 1: SPIRIT 2013 Checklist: Recommended items to address in a clinical trial protocol and related documents (DOC 121 kb)

## Data Availability

Individual-level de-identified patient data will be made publicly available after the study-specific aims have been published. The statistical analyses will be available for those who request it based on published analyses. Authorship of the final report will be based on contribution to the trial as determined by the principal investigators. The final report will be published in a peer-reviewed journal to facilitate communication to healthcare professionals and the general public. Published results will be shared with study participants should they indicate an interest in receiving this information (e.g., publications of these data will be sent as a pdf to their email address).

## Abbreviations

DNR: Do Not Resuscitate
EMPOWER: Enhancing & Mobilizing the POTential for Wellness & Emotional Resilience among Surrogate Decision-Makers of ICU Patients
EoL: End of life
HLM: Hierarchical linear modeling
ICU: Intensive care unit
PTSD: Post-traumatic stress disorder
RCT: Randomized controlled trial
